# The Parietal Eye of Lizards (*Pogona vitticeps*) Needs Light at a Wavelength Lower than 580 nm to Activate Light-Dependent Magnetoreception

**DOI:** 10.3390/ani10030489

**Published:** 2020-03-15

**Authors:** Tsutomu Nishimura

**Affiliations:** 1 Institute for Advancement of Clinical and Translational Science (iACT), Graduate School of Medicine, Kyoto University, 54 Kawahara-cho, Shogoin, Sakyo-ku, Kyoto 606-8507, Japan; tnishimu@kuhp.kyoto-u.ac.jp; 2 Translational Research Center for Medical Innovation, 1-5-4 Minatojima-minamimachi, Chuo-ku, Kobe 650-0047, Japan

**Keywords:** tail lifting, tail behavior, magnetoreception, magnetic sense, lizard, ELF-EMF, extremely low-frequency electromagnetic field

## Abstract

**Simple Summary:**

In this study, the author sought to identify the wavelength of light that activates light-dependent magnetoreception. *Pogona vitticeps* lizards were randomly divided into two groups. In both groups, small round light-absorbing filters were fixed to the back of each lizard’s head, to block light of wavelengths lower than 580 nm. The electromagnetic field group received 12 h of systemic exposure per day to an electromagnetic field at an extremely low frequency (light period), whereas the control group did not. For each animal, the average number of tail lifts per day was determined. No significant difference between the two groups, neither for the average ratio of the number of tail lifts on test days to the baseline value nor the average increase in the number of tail lifts on test days minus the baseline value (*p* = 0.41 and *p* = 0.67, respectively). The results of this experiment suggest that light-dependent magnetoreception in *P. vitticeps* only occurs when the light hitting the parietal eye is of a wavelength lower than 580 nm.

**Abstract:**

In a previous study, the agamid lizard *Pogona vitticeps* was discovered to respond to an electromagnetic field (EMF) of extremely low frequency (6 and 8 Hz; peak magnetic and electric fields of 2.6 µT and 10 V/m, respectively). Furthermore, when the third eye of a lizard was covered, using a small round aluminum cap, the reaction to the EMF disappeared. These results suggested that the parietal eye has a role in light-dependent magnetoreception. However, the wavelength of light needed to activate light-dependent magnetoreception has not been identified and was thus explored in the present study. Lizards were randomly divided into control and EMF groups. In both groups, a small round light-absorbing filter was positioned on the back of the head of each lizard and blocked light of wavelengths lower than 580 nm. The EMF group was subjected to EMF exposure for half of the day, whereas the control group was not. No significant intergroup differences were discovered in the average ratio of the number of tail lifts on test days to the baseline value or average increase in the number of test-day tail lifts minus the baseline value (*p* = 0.41 and *p* = 0.67, respectively). Lizards with light-absorption filters that cut out light with wavelengths lower than 380 nm were found to respond to the EMF. Therefore, the lizards appeared to respond to light of certain wavelengths rather than the filters themselves. The results of these experiments suggest that light of wavelengths lower than 580 nm is required to activate light-dependent magnetoreception in the parietal eye of *P. vitticeps*.

## 1. Introduction

Various animals, such as fish, turtles, mammals, birds, insects, and bacteria, have been reported to be sensitive to magnetism, which they use for orientation [1]. It has been reported that one lizard uses a geomagnetic sensor [2].

In our previous studies [3,4], we focused on tail-lifting behavior of lizards, including the agamid lizard *Pogona vitticeps* [5]. The lizard *Leiocephalus carinatus* shows tail-curling behavior during intraspecific agonistic encounters and courtship [6,7]. We discovered that exposure to electromagnetic fields (EMFs) of extremely low frequency (6 and 8 Hz; peak magnetic and electric fields, 2.6 µT and 10 V/m, respectively) increased the number of tail-lifts by lizards [3]. Additionally, when a small, round aluminum “cap” was employed to shield the parietal eye of each lizard, the reaction to the EMF disappeared. Some tuatara species (Rhynchocephalia) and lizards (Squamata) have been reported to have parietal eyes that are photosensitive [8]. Our results suggested a role of the parietal eye in light-dependent magnetoreception. However, the wavelength of light that activates this magnetoreception was not identified at that time. In the present study, an attempt was made to identify the wavelength of light that activates the light-dependent magnetoreception by evaluating the behavioral EMF responses of *P. vitticeps*.

## 2. Materials and Methods

The author obtained adult central bearded dragons (*P. vitticeps*; Agamidae) from a commercial source (Daiwa Pet Co., Kyoto, Japan) and bred the lizards for use in the experiment. Individual dragons were easily recognized according to their morphological characteristics. Four of the lizards used in Experiment 1 were used again in Experiments 2 and 3.

Birds are disoriented when exposed to wavelengths greater than 590 nm [9]. Therefore, as a first step, the author selected a filter that screened out light with wavelengths lower than 580 nm in Experiment 1/2. Since it has been reported that the photoreceptor cells of the lizard parietal eye are sensitive to blue and green light [10], filters having wavelengths lower than those were selected in Experiment 3.

The free-flying *Catharus* thrush (a night-migrating songbird) uses magnetic fields to determine its direction on very dark nights, even at light levels as low as 0.0003 lux [11]. In Experiments 1 and 2, and Experiment 3 in this study, the intensity of light entering the parietal eye was approximately 1000 and 500 lux, respectively. Therefore, the light intensity was sufficient to activate magnetoreception in animals. If the author adjusted the intensity of light entering the lizard’s parietal eye in Experiment 3, it was necessary to use twice as much light as that in Experiments 1 and 2. If the author used light that was twice as intense as that in Experiments 1 and 2, the light intensity that entered the lizards’ eyes was also twice as intense. The author believes that this is a considerable problem because doubling the light intensity may affect animal behavior; in this study, the intensity of light that entered the lizards’ eyes was the same as that in Experiments 1/2 and 3.

The Kyoto University Animal Research Committee approved all of the animal experiments (MedKyo09516).

### 2.1. Experiment 1

**Figure 1:**
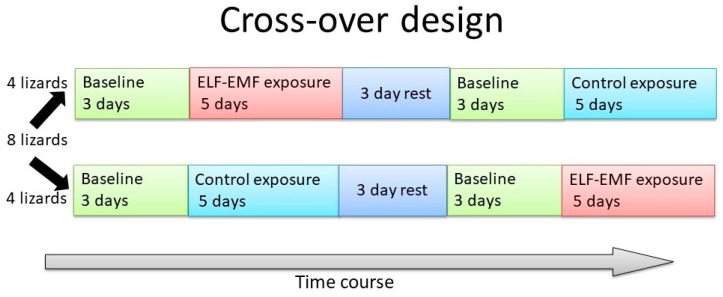
Overview of the Study design.

**Figure 2:**
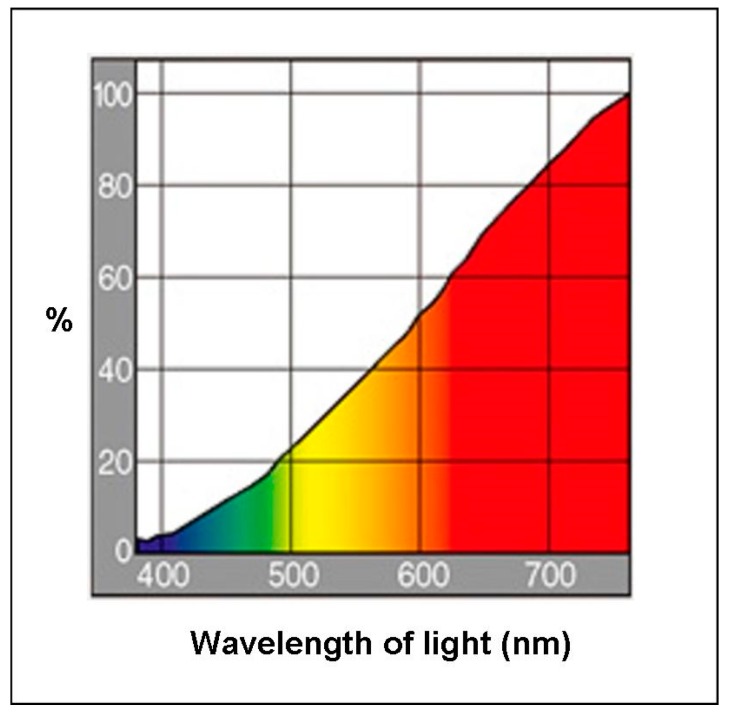
Spectral distribution of the incandescent light bulb (RS-100V 60W, Panasonic) (data from the producer’s catalogue).

This experiment was designed to determine whether the parietal eye of *P. vitticeps* requires light of wavelengths lower than 580 nm to activate light-dependent magnetoreception. A cross-over test was performed, using eight lizards (four males and four females; mean body weight (BW), 57.9 ± 40.4 g; mean snout–vent length (SVL), 10.4 ± 2.7 cm; mean total length (TL), 12.4 ± 6.1 cm) (Figure 1). Briefly, experiments were conducted twice; the control group in the first instance became the EMF group in the second instance, and the first EMF group served as the second control group. Between the two sessions in Experiment 1, the lizards had 3 days of rest. The lizards were housed in four terrariums (60 × 45 × 45 cm^3^), each divided in half by a wooden board (30 × 45 × 45 cm^3^) [3]. Each half of a terrarium had one lizard. Sheets of paper were fixed to the sides of each terrarium, half to prevent the lizards from seeing each other. Each terrarium housed one lizard each in the EMF and control groups. Four lizards in each group (eight in total) were simultaneously tested, and the experiment was performed twice. The four terrariums were stored in a single room that was maintained at a constant temperature and relative humidity (27.0 ± 1.5 °C and 50 ± 5%, respectively). Incandescent bulbs were used in this study and emitted a broad spectrum of light. A 12 h light–dark cycle was applied to the terrariums by using incandescent bulbs (RS-100V 60 W, Panasonic, Osaka, Japan, 2000 lux, lit at 09:00 h; Figure 2). The lizards had ad libitum standard food and tap-water access.

The definition of a tail lift and method of EMF exposure were identical to those used in another work [3]. The method of observing the number of tail lifts was the same as the method described as follows in the previous paper: “To monitor the number of tail lifts, images of each terrarium were captured automatically every minute for 24 h day-1 during the experimental period using two web cameras (CG-NCMNV2; Corega K.K., Kanagawa, Japan) placed in front of the terrariums and connected to a PC” [3].

**Figure 3:**
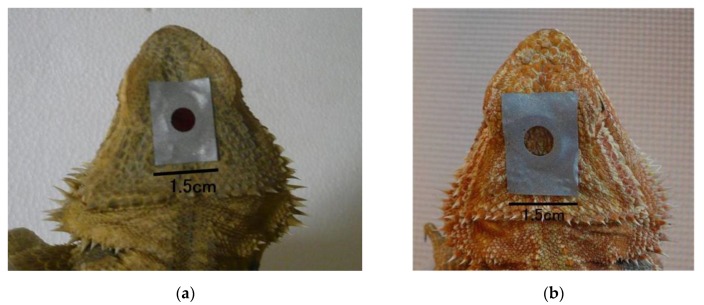
Small round light-absorption filter (6 mm in diameter) covering the parietal eye of *P. vitticeps* and blocking light of wavelengths (**a**) <580 nm and (**b**) <380 nm. The filter (**a**) was used in Experiment 1 and 2, and the filter (**b**) was used in Experiment 3.

**Figure 4:**
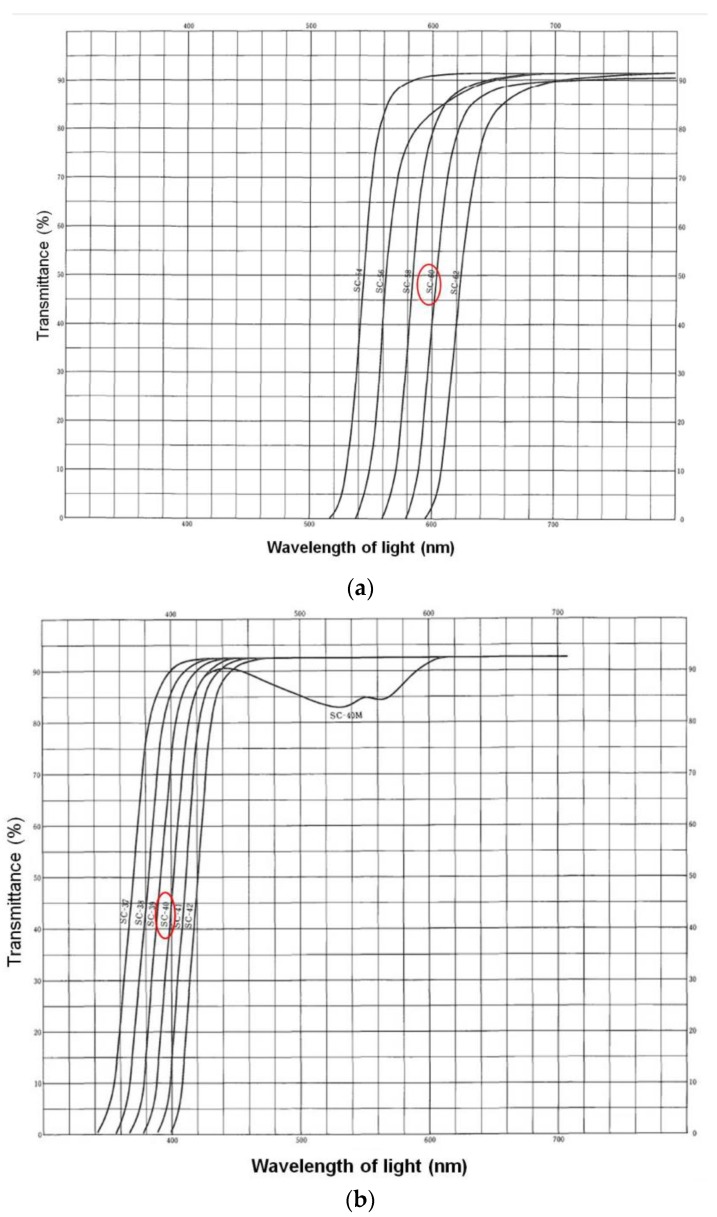
Transmittance of light-absorption filters (**a**) SC-60 and (**b**) SC-40. The filter (**a**) was used in Experiment 1 and 2, and the filter (**b**) was used in Experiment 3. Data from the Fujifilm photo handbook.

A small round light-absorbing filter (diameter of 6 mm, SC-60, Fujifilm Corporation, Tokyo, Japan; Figure 3a and Figure 4a) was attached to the dorsal surface of the heads of the lizards in both groups, by using tape and blocked light at wavelengths lower than 580 nm. These filters were retained during the baseline and exposure periods (3 and 5 days, respectively).

Experiment 1 was conducted from June 13 to July 1, 2009.

### 2.2. Experiment 2

The author confirmed the repeatability of the Experiment 1 findings by using eight adult lizards (four males and four females; average BW, 261.6 ± 44.4 g; average SVL, 16.9 ± 2.4 cm; average TL, 31.6 ± 5.1). Four of the lizards used in Experiment 1 were used again in Experiment 2. In the EMF group, the whole body of lizards was exposed to an EMF for 5 days for 11 h/day (08:00 to 19:00). The terrariums were subject to an 11/13 h light–dark cycle with incandescent light (RS-100V 60 W, Panasonic, ~2000 lux; light switched on at 08:00). The other details were identical to those for Experiment 1. Experiment 2 was conducted from June 15 to July 3, 2010.

### 2.3. Experiment 3

To determine whether attaching the light-absorption filter had an effect on tail-lifting behavior, Experiment 3 was performed, using the lizards from Experiment 2. In both groups, small round light-absorption filters (6 mm in diameter; SC-40, Fujifilm Corporation, Tokyo, Japan; Figure 3b and Figure 4b) that blocked light of wavelengths lower than 380 nm were attached, using tape, to the head’s dorsal surface for each lizard. These filters were retained during the baseline and exposure periods (3 and 5 days, respectively). The experiment was conducted from May 24 to June 11, 2010. Other details were identical to those in Experiment 2.

### 2.4. Statistical Analysis

The two sets of data used in the present cross-over study—corresponding to the set of data obtained for the lizards first in the control and then the EMF group and that obtained for the lizards in first the EMF group and then the control group—were merged and analyzed. Additionally, the Experiment 1 and 2 findings were combined. The Wilcoxon signed-rank test was applied to evaluate the intergroup difference in mean increase in test-day tail lifts minus the baseline value. SAS version 9.2 (SAS Institute, Cary, NC, USA) was employed for statistical analysis. Herein, values are expressed as the mean ± standard deviation (SD). A *p* < 0.05 denoted statistical significance.

## 3. Results

### 3.1. Experiment 1

No significant difference was discovered in the mean increase in tail lifts (test-day values minus baseline values) between the groups (1.5 ± 20.0 vs. 3.7 ± 18.4 in the control and EMF groups, respectively; *n =* 8 each, *p* = 0.66; Table 1). The intergroup difference in mean ratio of number of tail lifts (test-day values divided by baseline values) was also nonsignificant (1.1 ± 0.2 vs. 1.2 ± 0.2 in the control vs. EMF groups, respectively; *n =* 8 each, *p* = 0.45; Table 1).

### 3.2. Experiment 2

The mean increase in the number of tail lifts had a nonsignificant intergroup difference (−0.4 ± 3.7 vs. −0.4 ± 4.0 in the control and EMF groups, respectively; *n =* 8 each, *p* = 0.96; Table 1). No significant intergroup difference was found in the mean ratio of the number of tail lifts (0.9 ± 0.4 vs. 0.9 ± 0.3 in the control and EMF groups, respectively; *n =* 8 each, *p* = 0.96; Table 1).

### 3.3. Combined Data of Experiments 1 and 2

No significant intergroup difference was discovered in the mean increase in number of tail lifts (0.6 ± 14.3 vs. 1.6 ± 13.3 in the control and EMF groups, respectively; *n =* 16 each, *p* = 0.67; Table 1). The mean ratio of the number of tail lifts was also found to be not significantly different between the two groups (1.1 ± 0.2 vs. 1.2 ± 0.2 in the control and EMF groups, respectively; *n =* 16 each, *p* = 0.41; Table 1).

### 3.4. Experiment 3

A significant intergroup difference was discovered for the average increase in number of tail lifts (−0.5 ± 7.1 vs. 3.7 ± 9.4 in the control and EMF groups, respectively; *n =* 8 each, *p* = 0.02; Table 2). The control group had a significantly lower mean ratio of number of tail lifts than the EMF group (0.9 ± 0.3 vs. 1.9 ± 0.6, respectively; *n =* 8 each, *p* = 0.02; Table 1).

## 4. Discussion

In this study, the author attempted to identify the wavelengths of light necessary to activate light-dependent magnetoreception by using a light-absorbing filter that cut out light with wavelengths lower than 580 nm. No significant intergroup difference was discovered in terms of the average ratio of number of tail lifts on test days to baseline values and the average increase in number of tail lifts on test days from the baseline value. The finding’s reproducibility was confirmed by performing another cross-over study. In addition, an experiment was conducted in which a light-absorbing filter was used that cut out light with wavelengths lower than 380 nm, to determine whether the presence of the filter itself affected tail-lifting behavior. Both the average ratio and average increase in number of tail lifts exhibited significant differences between the two groups. Thus, light wavelength was the factor affecting the number of tail lifts rather than the presence of a filter on the heads of the lizards.

The mean number of daily tail lifts obtained for Experiment 1 was different from that obtained for Experiment 2. In Experiment 1, eight juvenile lizards were tested. They ate more food than the adult lizards, were more active, and did more tail lifts. The juvenile lizards used in Experiment 1 were also used in Experiment 2, at which point they had become adults. These factors are explanations for why more tail lifts were observed on average in Experiment 1. In Experiments 1 and 2, the lizards fitted with light-absorption filters that cut out light of wavelengths lower than 580 nm did not respond to the EMF, but in Experiment 3, the lizards with light-absorption filters that cut out light of wavelengths lower than 380 nm did respond to the EMF. Therefore, the lizards appeared to respond to light of a certain wavelength rather than the presence of a filter. The results of the present study thus strongly suggest a role of the parietal eye in light-dependent magnetoreception for lizards, a finding that has also been reported for salamanders [12,13]; the current findings also indicate that magnetoreception is only activated in the parietal eye of *P. vitticeps* when the incident light has a wavelength below 580 nm.

Table 2 shows increased tendency of tail lifts in EMF group over time on test days, but not in control group. In our previous study, we observed the P. vitticeps in EMF group and control group for two years and found that in the EMF group responded to the full moon, whereas those in the control group did not [4]. That is, the response to environmental factors may be different between the EMF group and the control group. There is the possibility that this difference influenced the difference in the tendency between the two groups on test days.

By using tail-lifting behavior in lizards as a magnetoreceptive reaction in our experiments, our experiments have a number of advantages: (1) Experiments can only be performed in certain seasons when using seasonal animals, but experiments can be performed at any time for lizards; (2) our experiments do not require specialized devices with which to cancel natural geomagnetic fields and generate artificial geomagnetic fields; and (3) the parietal eye can easily be shaded. For these reasons, our method may be helpful for researchers to study magnetoreception in animals.

Currently, researchers have proposed two hypotheses regarding magnetoreception: that it is a chemical compass that is driven by a radical-pair mechanism, and that it is a process involving magnetite particles [14]. According to the radical-pair model, magnetoreceptivity is dependent on light; the magnetic compass of birds and salamanders requires light [14]. The salamander *Notophthalmus viridescens* was observed to become disoriented in the absence of visible light [15]. In salamanders, light receptors were found in the pineal organ, as the third eye of ancient vertebrates was their pineal organ, which in amphibians have light sensitivity [16]. In a test, a colored filter was placed on the skull, above the pineal organ, but the eyes were exposed to natural light; the researchers discovered that the magnetic compass of the salamander was dependent only on the spectral characteristics of the light incident on the pineal organ [12,13]. Light has different effects on magnetic orientation in birds and newts. Orientation in birds occurs at light wavelengths lower than 590 nm [9]. Salamanders trained in the heading direction exhibited the normal orientation only when exposed to light of wavelength 450 nm or lower. When exposed to light with a wavelength of 475 nm, they were disoriented. At wavelengths greater than 500 nm, their orientation was shifted approximately 90° counterclockwise [13]. Thus, the current results are consistent with those obtained for birds but not for salamanders. This inconsistency can be explained by birds and *P. vitticeps* being diurnal but salamanders being nocturnal.

## 5. Conclusions

In summary, the results of the present experiments suggest that light-dependent magnetoreception is activated in the parietal eye of *P. vitticeps* only for light with wavelengths less than 580 nm.

## Figures and Tables

**Table 1:** Combined data from Experiments 1 and 2 (wavelengths < 580 nm were filtered out, cross-over study; control group vs. electromagnetic field (EMF) of extremely low frequency group, *n =* 16 each): mean number of tail lifts, ratio of the number of tail lifts (test-day values divided by baseline values), and increase in number of tail lifts (test-day values minus baseline values; data shown are means ± SD per individual per day).

**Experiments 1 and 2**	Control Group *			EMF Group *		
	**No. of Tail Lifts**	**Ratio ^a^**	**No. of Increase ^b^**	**No. of Tail Lifts**	**Ratio ^a^**	**No. of Increase ^b^**
Pre-test baseline	9.4 ± 14.6	1	0	9.8 ± 15.3	1	0
Test-day values						
Day 1	9.5 ± 14.4	1.0	0.1 ± 6.9	13.8 ± 20.7	1.4	4.0 ± 9.7
Day 2	9.8 ± 20.7	1.0	0.4 ±10.5	11.7 ± 16.7	1.2	1.9 ± 13.8
Day 3	9.1 ± 12.6	1.0	−0.3 ± 11.5	10.6 ± 14.8	1.1	0.7 ± 14.8
Day 4	9.0 ± 16.0	1.0	−0.4 ± 15.0	11.4± 16.8	1.2	1.5 ± 17.7
Day 5	12.4 ± 25.6	1.3	3.0 ± 23.7	9.9 ± 12.4	1.0	0.0 ± 10.6
Days 1–5 combined	10.0 ± 18.0	1.1 ± 0.2	0.6 ± 14.3	11.5 ± 16.1	1.2 ± 0.2	1.6 ± 13.3
**Experiment 1**	Control Group *			EMF Group *		
	**No. of Tail Lifts**	**Ratio**	**No. of Increase**	**No. of Tail Lifts**	**Ratio**	**No. of Increase**
Pre-test baseline	15.5 ± 18.5	1	0	16.5 ± 18.8	1	0
Test-day values						
Day 1	16.6 ± 18.0	1.1	1.1 ± 9.8	24.4 ± 25.1	1.5	7.9 ± 12.6
Day 2	16.3 ± 28.4	1.0	0.7 ± 14.8	20.4 ± 20.4	1.2	3.9 ± 19.7
Day 3	13.5 ± 16.3	0.9	−2.0 ± 15.6	20.1 ± 15.9	1.2	3.7 ± 20.8
Day 4	16.4 ± 20.5	1.1	0.8 ± 21.5	18.8 ± 21.3	1.1	2.3 ± 25.3
Day 5	22.5 ± 34.1	1.5	7.0 ± 33.9	17.1 ± 14.0	1.0	0.7 ± 15.1
Days 1–5 combined	17.1 ± 23.3	1.1 ± 0. 2	1.5 ± 20.0	20.2 ± 18.9	1.2 ± 0.2	3.7 ± 18.4
**Experiment 2**	Control Group *			EMF Group *		
	**No. of Tail Lifts**	**Ratio**	**No. of Increase**	**No. of Tail Lifts**	**Ratio**	**No. of Increase**
Pre-test baseline	3.3 ± 3.5	1	0	3.2 ± 5.6	1	0
Test-day values						
Day 1	2.4 ± 1.6	0.7	−0.9 ± 1.6	3.3 ± 5.4	1.0	0.0 ± 2.7
Day 2	3.4 ± 3.7	1.0	0.1 ± 4.1	3.0 ± 3.2	0.9	−0.2 ± 3.3
Day 3	4.8 ± 5.7	1.4	1.5 ± 5.7	1.0 ± 1.9	0.3	−2.2 ± 3.8
Day 4	1.6 ± 2.8	0.5	−1.7 ± 3.6	4.0 ± 4.9	1.3	0.8 ± 5.0
Day 5	2.4 ± 2.4	0.7	−0.9 ± 4.1	2.6 ± 3.4	0.8	−0.6 ± 3.7
Days 1–5 combined	2.9 ± 3.5	0.9 ± 0.4	−0.4 ± 3.7	2.8 ± 3.9	0.9 ± 0.3	−0.4 ± 4.0

* Two datasets (that for EMF grouping, followed by control grouping, and that for control grouping, followed by EMF grouping) were combined and analyzed. ^a^ The ratio of test-day values divided by the baseline values. ^b^ The increase in the number of tail lifts = test-day values minus baseline values.

**Table 2:** Experiment 3 results (cross-over study; filtering out light of wavelengths < 380 nm; control group vs. EMF group, *n =* 8 each): average number of tail lifts, ratio of the number of tail lifts (test-day values divided by baseline values), and increase in the number of tail lifts (test-day values minus baseline values; data shown are mean ± SD per individual per day).

Experiment 3	Control Group *			EMF Group *		
	No. of Tail Lifts	Ratio ^a^	No. of Increase ^b^	No. of Tail Lifts	Ratio ^a^	No. of Increase ^b^
Pre-test baseline	5.9 ± 6.3	1	0	4.0 ± 4.7	1	0
Test-day values						
Day 1	3.8 ± 4.1	0.6	−2.2 ± 6.1	4.1 ± 4.1	1.0	0.1 ± 5.9
Day 2	7.1 ± 9.1	1.2	1.2 ± 8.9	7.1 ± 9.9	1.8	3.1 ± 10.6
Day 3	7.4 ± 5.7	1.2	1.5 ± 5.3	7.9 ± 9.0	2.0	3.9 ± 10.4
Day 4	4.3 ± 2.8	0.7	−1.7 ± 6.2	8.8 ± 11.2	2.2	4.8 ± 10.5
Day 5	4.9 ± 6.8	0.8	−1.6 ± 9.2	10.6 ± 11.8	2.6	7.1 ± 10.1
Days 1–5 combined	5.5 ± 5.9	0.9 ± 0.3	−0.5 ± 7.1	7.6 ± 9.3	1.9 ± 0.6	3.7 ± 9.4

* Two sets of data (that for EMF grouping followed by control grouping and that for control grouping followed by EMF grouping) were merged and underwent analysis. ^a^ Ratio = number of tail lifts divided by the baseline number of tail lifts (0.9 ± 0.3 vs. 1.9 ± 0.6 in the control and EMF groups, respectively; *n =* 8 each, *P* = 0.02). ^b^ Increase in the number of tail lifts = test-day value minus baseline value (−0.5 ± 7.1 vs. 3.7 ± 9.4 in the control and EMF groups, respectively; *n =* 8 each, *p* = 0.02).
